# Sequence Imputation of HPV16 Genomes for Genetic Association Studies

**DOI:** 10.1371/journal.pone.0021375

**Published:** 2011-06-23

**Authors:** Benjamin Smith, Zigui Chen, Laura Reimers, Koenraad van Doorslaer, Mark Schiffman, Rob DeSalle, Rolando Herrero, Kai Yu, Sholom Wacholder, Tao Wang, Robert D. Burk

**Affiliations:** 1 Department of Pediatrics, Albert Einstein College of Medicine, Bronx, New York, United States of America; 2 Department of Microbiology and Immunology, Albert Einstein College of Medicine, Bronx, New York, United States of America; 3 Department of Obstetrics, Gynecology and Women's Health, Albert Einstein College of Medicine, Bronx, New York, United States of America; 4 Division of Cancer Epidemiology and Genetics, National Cancer Institute, Bethesda, Maryland, United States of America; 5 Sackler Institute of Comparative Genomics, American Museum of Natural History, New York, New York, United States of America; 6 Proyecto Epidemiológico Guanacaste, Fundación INCIENSA, San José, Costa Rica; 7 Department of Epidemiology and Population Health, Albert Einstein College of Medicine, Bronx, New York, United States of America; British Columbia Centre for Excellence in HIV/AIDS, Canada

## Abstract

**Background:**

Human Papillomavirus type 16 (HPV16) causes over half of all cervical cancer and some HPV16 variants are more oncogenic than others. The genetic basis for the extraordinary oncogenic properties of HPV16 compared to other HPVs is unknown. In addition, we neither know which nucleotides vary across and within HPV types and lineages, nor which of the single nucleotide polymorphisms (SNPs) determine oncogenicity.

**Methods:**

A reference set of 62 HPV16 complete genome sequences was established and used to examine patterns of evolutionary relatedness amongst variants using a pairwise identity heatmap and HPV16 phylogeny. A BLAST-based algorithm was developed to impute complete genome data from partial sequence information using the reference database. To interrogate the oncogenic risk of determined and imputed HPV16 SNPs, odds-ratios for each SNP were calculated in a case-control viral genome-wide association study (VWAS) using biopsy confirmed high-grade cervix neoplasia and self-limited HPV16 infections from Guanacaste, Costa Rica.

**Results:**

HPV16 variants display evolutionarily stable lineages that contain conserved diagnostic SNPs. The imputation algorithm indicated that an average of 97.5±1.03% of SNPs could be accurately imputed. The VWAS revealed specific HPV16 viral SNPs associated with variant lineages and elevated odds ratios; however, individual causal SNPs could not be distinguished with certainty due to the nature of HPV evolution.

**Conclusions:**

Conserved and lineage-specific SNPs can be imputed with a high degree of accuracy from limited viral polymorphic data due to the lack of recombination and the stochastic mechanism of variation accumulation in the HPV genome. However, to determine the role of novel variants or non-lineage-specific SNPs by VWAS will require direct sequence analysis. The investigation of patterns of genetic variation and the identification of diagnostic SNPs for lineages of HPV16 variants provides a valuable resource for future studies of HPV16 pathogenicity.

## Introduction

**Figure 1:**
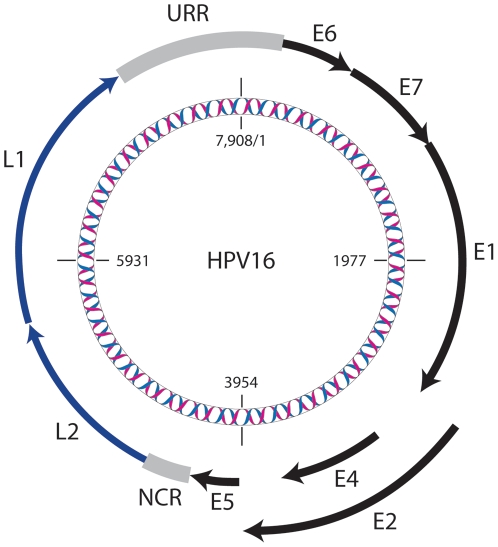
A schematic representation of the HPV16 genome. The 7,908 bp circular, double-stranded DNA genome of the HPV16 reference sequence is illustrated. Genes expressed early in the viral life cycle (early genes) are drawn with solid black lines and their names prefixed by an “E”. Genes expressed late in the viral life cycle (late genes) that encode the structural capsid proteins, are indicated by blue lines and have names prefixed by an “L”. The upstream regulatory region (URR) and a second non-coding region (NCR) are drawn in gray. The viral genome and positions within the DNA sequence are displayed by the helix and numbers, respectively.

Human papillomaviruses (HPVs) are a highly prevalent, globally distributed group of DNA viruses infecting cutaneous and mucosal epithelia throughout the human body [1], [2]. HPV type 16 (HPV16) is the most potent carcinogen and the most studied of the HPVs [3], [4]. Persistent infection with high-risk HPV is responsible for over 90% of invasive cervical cancers worldwide; HPV16 accounts for approximately two thirds of the cervical cancers [3], [4] and up to 90% of HPV-associated extra-cervical tumors [5]. HPVs contain a 7.9-kb circular double-stranded DNA genome that consists of four parts: an early region, a late region, an upstream regulatory region (URR) and a small, highly variable, non-coding region (NCR) between E5 and L2 (figure 1). All known HPV types, of which there are over 150, have nearly identical gene content and organization. An individual HPV type is defined based on the cloned genome being at least 10% different in the L1 open reading frame nucleotide sequence from all other characterized HPV types; variants are isolates with less than 10% sequence diversity [1], [6]. Collectively, HPVs show a range of tissue tropisms and associated pathologic manifestations such as skin warts, respiratory papillomatosis, condyloma acuminata and cervix cancer, all associated with different evolutionarily related HPV genomes [1], [6].

Genetically, papillomaviruses (PVs) evolve slowly, with a mutation rate of approximately 2±0.5×10^−8^ per nucleotide per year [7]. They are predominantly host- and tissue-specific, with HPV16 displaying Darwinian selection at a limited set of codon sites across the whole genome [8]. In addition, PVs show little, if any, definitive recombination throughout their evolutionary history. Consequently for HPV genetics, nucleotide polymorphisms have occurred through random mutation and subsequently become fixed within a small number of viral lineages, within each type, over evolutionary time [8], [9], [10]; for HPV16, variant lineages are currently named according to population groups in which they are most prevalent (see [1], [11], [12] for definitions). Current knowledge of HPV and cervix oncogenesis is primarily based on the association of categorically classified high-risk (HR) HPV types [3], [4], [13] and in some cases variant lineages of HPV types (e.g., HPV16 non-European variant lineages) [10]. However, to advance understanding of the genetic basis of HPV-associated carcinogenesis, it is anticipated that complete sequencing of many hundreds to thousands of HPV genomes from large population-based and case-control studies of cervix neoplasia and cancer will be required [14].

Genotype imputation relies on a high correlation between genetic variants at sites across the genome of an organism (for review see [15]). In genome-wide association studies (GWAS), imputation can improve the coverage of genotyping arrays [15], [16], [17], which only measure a small proportion of genetic variation in a study sample. Typically, a subset of single nucleotide polymorphisms (SNPs) from individuals in a study population is assayed for association with a particular disease or phenotypic trait. It is possible to use partial genotype data together with information about shared stretches of DNA (e.g., linkage disequilibrium (LD) data available from the HapMap project) to detect associations between unmeasured SNPs and a given disease or phenotype [15]. In contrast, lineage fixation of DNA polymorphisms within the HPV genome leads to the possibility of imputing missing genomic sequences from short regions using the growing assembly of diverse complete HPV16 genome sequences [8].

Although the extraordinarily high association (odds ratio >300) between HPV16 and cervical cancer [18] is strictly related to the DNA content of the HPV16 genome, the specific underlying genetic basis of the association is unknown. To address this, we performed two analyses. The first assessed HPV16 phylogeny and patterns of genetic variation from a reference set of complete genome HPV16 sequences covering all known lineages. In studying these, we identified diagnostic SNPs across the genome for all major lineages of HPV16. The second investigated the use of imputed SNPs in a viral genome-wide association study (VWAS) from a set of HPV16 sequenced fragments from the URR/E6 region. In order to obtain complete genomes from the partial genomic data, we developed and tested an automated method to impute missing nucleotides using a reference database, stand-alone BLAST+ [19] and a custom BLAST output parser written in Mathematica 7.0 (Wolfram Research, Inc., Mathematica, Version 7.0, Champaign, IL, 2008). This methodology was found more suitable for HPV than other currently existing imputation software (e.g., fastPHASE [20] and IMPUTE [21]). All SNP sites identified, including diagnostic SNPs, were then tested for association with disease outcome (i.e., histologically confirmed cervical intraepithelial neoplasia 3 and cancer (CIN3+)). The analyses presented here focused on the HPV16 positive patients in the HPV Natural History Study in Guanacaste, Costa Rica [10], indicating the feasibility of future large scale VWASs in HPV-associated cancer. This report reveals a new appreciation of HPV16 sublineages as risk determinants.

## Results

### Inter-sequence identity and phylogeny

A global alignment (available from authors upon request) of HPV16 complete genomes was performed to examine patterns of nucleotide variation. The panel consisted of 62 complete genome sequences (Table S1) containing isolates representing the majority of known HPV16 variant lineages (for NCBI accession numbers see Supplemental Table S1). The resulting alignment was 7916 bp and introduced a maximum of 10 insertions/deletions (indels) across all sequences. All indels were within the highly variable non-coding region between E5 and L2 (NCR, positions 4102–4236, see Fig. 1) of the HPV16 genome. A pairwise comparison between all sequences identified a total of 540 single nucleotide polymorphisms (SNPs) with any pair of sequences having a maximum of 180 (2.3% of genome) differences.

**Figure 2:**
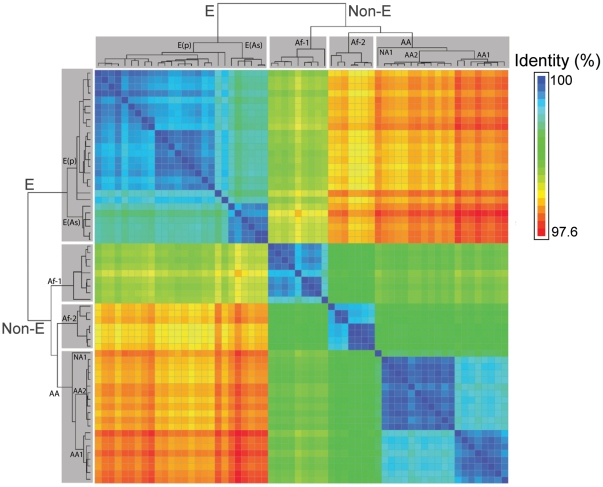
HPV16 phylogeny and heatmap of HPV16 isolates in the reference panel. All 62 nucleotide sequences in the HPV16 complete-genome reference panel were aligned and compared to one another. Sequence identity between every pair was measured and represented as a heatmap, scaled such that the minimum inter-sequence identity (97.6%) is displayed as red and the maximum inter-sequence identity (100%) as blue. A Bayesian phylogenetic tree is shown alongside the heatmap to illustrate how the inter-sequence identities relates to the phylogenetic topology. Major lineages (highlighted in gray) and sublineages are labeled and all have 100% bootstrap support.

Prior phylogenetic analyses of HPV16 variants [8], [22], [23], [24] identified 4 major intratypic variant lineages: European (E), Asian-American (AA), African-1 (Af-1) and African-2 (Af-2). Figure 2 depicts the whole-genome Bayesian phylogenetic tree and a heatmap of the pairwise identities between all HPV16 complete-genome sequences in the reference panel (Fig. 2). In addition to the differences between the major variants, there appears to be sublineage structure; a feature notably well developed within the AA lineage. The tree demonstrates excellent support (i.e., all partitions have posterior probability of 1.0) for the placement of all named lineages and sublineages (Chen et al., manuscript in preparation); E, Non-E, European prototype (E(p)), European Asian (E(As)), Af-1, Af-2, AA, North American (NA1), Asian-American 1 and 2 (AA1 and AA2). Where, by “sublineages” we refer to groups of sequences with 0.5% –1.0% differences between their genomes [25]. Sublineages appear as distinct blocks of similar color in Figure 2. Taken together, these analyses suggest the presence of evolutionarily fixed sublineages at a deeper taxonomic level than the 4 commonly recognized variant clades (i.e., E, AA, Af-1 and Af-2) [8].

### Analysis of genetic variation

**Figure 3:**
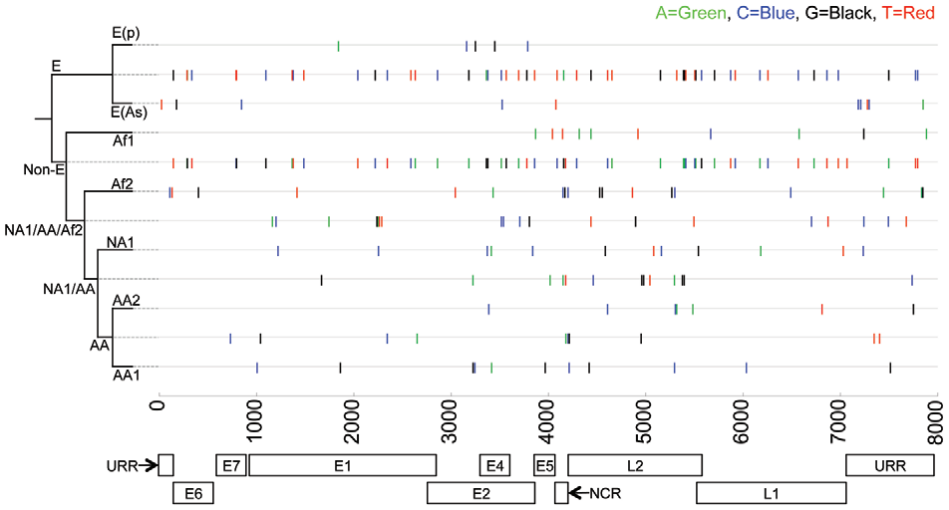
Lineage-specific single nucleotide polymorphisms (SNPs) in HPV16 variants and their position in the genome. Lineage-specific SNPs were determined from an alignment of the 62 complete-genome nucleotide sequences by selecting the nucleotides that occurred only in members of a given lineage. The value of each SNP is color-coded as shown at the top right of the figure. The SNPs are plotted by position in the HPV16 genome on the *x*-axis and aligned according to lineage in the phylogenetic tree on the *y*-axis. SNPs for a given lineage are cumulative as the tree is traversed from deepest node out to branches. Thus, for example, HPV16 genomes of Af-2 lineage (row 6) contain all SNPs shown on the Non-E (row 5), AA/NA1/Af-2 (row 7) and Af-2 (row 6) lines. Regions of the genome are displayed below the *x*-axis for reference.

To evaluate the ability to use sequence information from limited, specific regions of the HPV16 genome for imputation, we had to determine the correlation of SNPs between regions. Due to the non-recombinant nature of HPV, diagnostic (i.e., lineage-specific) SNPs could be identified and plotted against position in the genome for each separate phylogenetic lineage (Fig. 3). Of note, non-lineage-specific SNPs appear in some genomes and not others with a pattern independent of lineage (e.g., T350G appears in some E and all characterized AA genomes). The analysis showed that any given fragment of the genome ≥500 bp in size would contain SNPs enabling discrimination of E from Non-E (see Fig. 3, rows 2 and 5). At deeper nodes within the phylogenetic tree, some lineages showed diagnostic SNPs that are only present within a particular region of the genome. For example, the Af-1 lineage (Fig. 3, row 4) has no diagnostic SNPs in the first 3900 bp of the genome, thus most of this region could not be used to assign a lineage of Af-1(see Fig. 3). This indicates that specific regions of the genome differ in their capacities for lineage assignment.

The L1, E6 and/or the URR regions of the HPV16 genome are frequently utilized for variant studies. Typically, epidemiological studies assign HPV16 lineage based upon the nucleotide sequence of one or more of these regions [26], [27]. The SNP distribution analysis (Fig. 3) revealed that the URR region contained sufficient diagnostic SNPs to discriminate between all distinct sublineages: E(p), EAs, Af-1, Af-2, NA1, AA1 and AA2. Thus, SNPs that occur within the URR are coincident with other lineage defining SNPs that occur across the rest of the genome. This observation indicates that imputation based on matching partial regions by sequence identity would assign a sequence from the correct sublineage when the URR region was known. In addition to the URR, the E2/E4 overlap region was found to provide discriminatory power, through combinatorial sets of SNPs, between all identified HPV16 sublineages despite containing no unique diagnostic SNPs for the AA and Af-1 lineages (Fig. 3).

The L1 ORF was found to allow discrimination between all but the E(As)/E(p) sublineages. By contrast, the E6 ORF was able to resolve the two European sublineages (E(p) vs. E(As)), and the African sublineages (Af-1 vs. Af-2), but unable to distinguish the Asian-American sublineages (NA1 vs. AA1 vs. AA2). The E6 and L1 ORFs, individually, provide limited information on viral lineage. In summary, the URR and E2/E4 overlap region contain diagnostic SNPs that can be genotyped to determine HPV16 viral lineage. All diagnostic SNPs (plotted in Figure 3) are listed in Table S2.

### Imputation

The first stage of the imputation procedure, search of the partial-genome query sequences (i.e., previously determined URR/E6 fragments [10]) against the database of complete HPV16 genomes by BLAST, identified several best matches for many of the query sequences amongst the complete-genome library (mean number of equally high scoring BLAST hits per partial sequence  = 6.8±2.7 (s.d.)). This indicated that sequence information from the genome fragments was not always sufficient to uniquely determine the exact genotype from amongst the group of HPV16 reference sequences. To address this issue, we randomly sampled from the pool of equally well matching complete genome sequences 100 times to produce multiple imputations that could be used in further analyses (see Materials and Methods).

### Characterizing imputation error rate

The accuracy of sequence imputation was estimated using three different methods: imputation using the URR sequence (833 nucleotides) from each of the 62 complete genomes in the reference database with and without removal of the test genome; and, complete-genome sequencing of 8 previously unsequenced, randomly selected samples from amongst the imputed study samples (for NCBI accession numbers see Supplemental Table S1). Imputation with removal of the test genome was used to estimate the error introduced by imputation when the true sequence was not present in the reference panel. The mean estimated proportion of correctly imputed SNPs in this case was 527.5±2.4 out of 540 (97.68±0.45%) SNPs. Testing imputation without removal of the true genome sequence estimated the possible error introduced when the true sequence was present in the reference panel. Error in this scenario is introduced when matches are identical in the test region (e.g., the URR), but different across the rest of the genome. The mean estimated proportion of correctly imputed SNPs under these conditions was 537.3±1.9 out of 540 (99.51±0.35%) SNPs. Complete-genome sequencing was performed on a randomly selected subset of 8 samples containing HPV16 genomes not previously sequenced. This method showed that a mean of 527.0±5.5 out of 540 (97.52±1.03%) of known SNPs in the sample population were imputed correctly.

### Case-control HPV16 SNP association analysis

**Figure 4:**
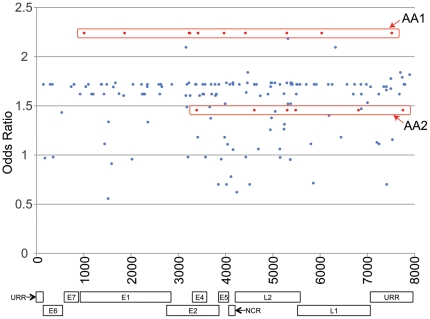
HPV16 viral genome-wide association study (VWAS). Odds ratios for an outcome of CIN3+ vs. resolved HPV16 infections were calculated for every SNP that occurred >10 times in the imputed and determined nucleotide sequences for 412 HPV16 positive samples from the Guanacaste, Costa Rica cohort [10]. Odds ratios for each SNP are plotted against their position in the genome. Some stratification of SNPs by odds ratio is evident. By mapping the bands of SNPs back to the lineage-specific SNP plot, it was possible to determine that the band with the highest odds ratio was composed of SNPs from the AA1 sublineage. For comparison, red boxes indicate the bands containing AA1- and AA2-specific SNPs. Due to statistical power limitations in this study, none of the SNPs attained viral genome-wide significance. Despite this, the plot suggests differences in disease association between sublineages.

To evaluate the association of all known SNPs amongst HPV16 variants with an outcome of CIN3+ compared to the HPV16 infections that resolved, a plot of odds ratios against SNP position in the genome was performed for the imputed data sets (Fig. 4). The plot produced a pattern of stratified odds ratios, rather than demonstrating a small number of high odds ratio SNPs. The highest “stratum” was occupied, upon inspection, by the diagnostic SNPs of a distinct sublineage (AA1), suggesting a higher risk for CIN3+ (see top red box in Fig. 4). Overall odds ratio calculations showed an increased association of the AA lineage with CIN3+, OR  = 1.73 (p = 0.07, 95% C.I. 0.95–3.18). Analyzed individually, the AA1 sublineage was found to have an odds ratio of 2.24 (p = 0.16, 95% C.I. 0.74–6.81) and the AA2 sublineage, an odds ratio of 1.46 (p = 0.30, 95% C.I. 0.71–2.97), corresponding to the odds ratios of their lineage-specific SNPs shown in Fig. 4 (see both red boxes). Further analysis revealed that all lineages occupied their own strata (i.e., lineage-specific SNPs for a given lineage all had equal odds ratios), consistent with the lineage fixation of SNPs in the HPV genome [8]. Non-lineage-specific SNPs yielded a range of odds ratios between 0 and 2.35 (that of the AA1 lineage). Although none of the SNPs' odds ratios in Figure 4 reached statistical significance, this analysis did provide novel insights not anticipated prior to the analysis. In particular, the possibility that a specific sublineage (AA1) may be driving the increased odds ratio of pooled Non-European variants was a surprise and has broad implications.

## Discussion

The association of HPV and cervix cancer has always been based on the detection of the HPV genome, since standard virologic methods proved insensitive [28]. The association of HPV genomes with cervix cancer was first established prior to the development of PCR when only crude assays were available and there was limited ability to distinguish individual HPV types [29]. With the development and improvement of HPV assays, it became well recognized throughout the world that association with cervix cancer risk was based on the DNA sequence of the HPV genomes, with the genome of HPV16 having an extraordinary association with cervix cancer [18]. Other evolutionarily related HPV genomes, predominantly of the alpha-9 (HPV16-related) and alpha-7 (HPV18-related) species groups, were also shown to be associated with cervix cancer, but less so than HPV16. Nevertheless, the underlying nucleotide changes responsible for the association with cancer and more specifically, the huge risk associated with HPV16, have gone largely unsolved. Further developments in DNA sequencing technology allowed a finer resolution of HPV types into variant lineages, and application of sequencing short regions of the HPV genome provided compelling evidence that variants of HPV16 of the non-European lineage had a stronger association with high-grade cervix neoplasia and cancer than the European lineage [10], [30], [31]. This report is a step forward in understanding the genetic basis of HPV carcinogenicity at the nucleotide level. We assessed sequence imputation from a large number of complete genome sequences using knowledge about the evolution of HPV16 genomes [8] and demonstrate that lineage fixation is so strong that diagnostic SNPs for evolutionarily stable variant sublineages can be found and are distributed across the genome. We observed a subgroup of an HPV16 AA variant lineage with a possibly elevated risk for CIN3+ within this host population. This latter observation suggests that future work might be able to stratify a virus carrier's risk of precancer or cancer by different lineages, sublineages and possibly even smaller evolved groupings.

At the nucleotide level, we show that the fixation of most SNPs within lineages allows imputation of the conserved lineage specific SNPs with high accuracy; however, the majority of imputed SNPs do not provide new information on risk assessment, since they are highly correlated. Causal SNPs that happen to be lineage-specific cannot be distinguished from non-causal lineage-specific SNPs, based on odds ratio. This is due to the high lineage fixation (equivalent to linkage disequilibrium in recombining genomes) in HPV16 and means that, as performed here, phylogenetic information must be taken into account when analyzing SNPs with elevated odds ratios in HPV. SNPs within high LD (non-recombining) regions of the human and other diploid genomes will have similar properties. In addition, the set of non-lineage-specific SNPs, those that are not correlated with the set of measured SNPs, will have to be determined directly before their contribution to risk and pathogenesis can be assessed.

The analysis of genetic relatedness (Fig. 2) between the complete genomes of a large set of HPV16 isolates, sampled for maximum diversity from around the world, demonstrated the presence of distinct and evolutionarily stable sublineages. Analysis of lineage defining SNPs in the HPV16 genome revealed that for many nodes on the phylogenetic tree, diagnostic SNPs were distributed across the entire genome, in contrast to genomes that undergo recombination with SNPs typically being correlated by distance [32]. Most publicly available imputation software (e.g., fastPHASE [20], IMPUTE [21], PLINK [33], EMINIM [16]) requires pedigree information or large-scale reference panels of haplotypes with information on several million SNPs, as well as recombination and mutation parameters which are pre-defined or extrapolated from the data. Hidden Markov Models, upon which this type of software is based, are suited to imputing where typed (measured) markers are distributed across the entire genome and are useful for genomes that undergo recombination. Additionally, because most genetic studies focus on diploid organisms, few of the publicly available programs are designed to work with other types of genomes (e.g., double stranded DNA viral genomes such as HPV). These software (i.e., fastPHASE and IMPUTE) were evaluated for use with our data, but it was ultimately determined that a simple BLAST-based approach would more effectively leverage the properties of our data and HPV genetics, and simplify interpretation of results.

Imputation error estimation showed that imputation produced only a small number of erroneously assigned SNPs per sequence (<20, corresponding to <0.25% of the genome and <3.7% of all known SNP positions in the reference panel). Simulation of imputation without removal of the test genome was likely an overestimate of the accuracy, but was used to account for multiple closely related matches. Simulation of imputation with removal of the test genome, by contrast, may have underestimated the mean accuracy, since a proportion of the query samples came from lineages over-represented in the reference panel. Errors, under the former circumstances, occur when multiple sequences in the reference panel are identical over the region covered by the partial sequence information but different across the remaining genome. As the database of complete HPV16 genomes expands to cover essentially all recurring variations throughout the world, it can be estimated that imputation accuracy will reach a maximum, close to but not equal to 100%. There will always be a set of SNPs (n ≈ 20) that are variable within and between lineages (e.g., the E6 variable position T350G) that do not allow absolute imputation, these HPV16 SNPs will require direct determination to evaluate their risk for cancer.

We used the genome sequences of 412 HPV16 infected cases and controls (396 imputed and 16 sequenced) to perform a VWAS to identify SNPs that confer additional risk for developing high-grade cervical intraepithelial neoplasia. Although this low-power ‘proof of principle’ analysis found no statistically significant individual SNPs, we identified intriguing trends at the sublineage level. In this dataset, there is a higher association with CIN3+ for Non-E lineages than for E lineages [10]. Figure 4 and the subsequent categorical odds ratio calculation suggest that the elevated association of the AA1 sublineage may drive the elevated risk of the Non-E lineage. Morevoer, the AA1 sublineage is characterized by the presence of just 11 SNPs. This opens the door to trying to verify lineage-based risk stratification. It also raises the question whether or not the effect is population specific. If confirmed, analysis of these 11 SNPs could narrow down the list of possible mechanisms that are driving differences in pathogenicity. Further studies with larger populations will be required to determine the validity of this putative association and whether risk can be further stratified to more distal lineages.

## Materials and Methods

### Epidemiological data

Case and control state was obtained from the large population-based cohort study (10,049 women) conducted in Guanacaste, Costa Rica, previously described [34], [35]. Of the 10,049 participants, 412 who were positive for HPV16 (HPV testing methods described in [8], [36], [37], [38]) and designated as either case (patient developed histologically confirmed cervical intraepithelial neoplasia 3 or cancer (CIN3+)) or control (patient had HPV16 detected but infection resolved) were selected for use in the viral genome-wide association study (VWAS) proof of principle analysis. Costa Rican and National Cancer Institute of the United States institutional review boards approved all study protocols. All participants signed an informed consent form. The study was also approved by the Committee on Clinical Investigation at the Albert Einstein College of Medicine.

### HPV16 DNA sequencing

DNA isolated from exfoliated cervical cell samples was used for all HPV analyses, including initial HPV detection, typing and variant sequencing, as previously described [8], [10], [39], [40]. HPV16 genome sequences and the NCBI/GenBank accession numbers are listed in Table S1 in the supplemental material. There were 16 complete-genome sequences and 396 partial sequences from the nested case-control study. Of the 412 cases and controls, 115 were cases (CIN3+) and 297 were controls (HPV16 infections that spontaneously cleared).

### Sequence alignment and phylogenetic analysis

Complete-genome sequences of HPV16 (n = 62, Supplemental Table S1) were aligned using CLUSTAL W [41]. Nucleotide positions are given relative to the HPV16 reference sequence [42]. A Bayesian tree inferred from the alignment of these 62 sequences was constructed using the Markov Chain Monte Carlo (MCMC) algorithm in MrBayes v3.1.2 with 10,000,000 cycles, where the first 1,000,000 cycles were discarded [43], [44]. The computer program ModelTest v3.7 [45] was used to identify the best evolutionary model; the identified gamma model was set for among-site rate variation and allowed substitution rates of aligned sequences to be different.

### Heatmap plot and pairwise identity analysis

Pairwise identity analysis between all 62 complete-genome sequences was performed in Mathematica 7.0 (Wolfram Research, Inc., Mathematica, Version 7.0, Champaign, IL, 2008; see Supplemental Data S1 for code). A ClustalW alignment of all (*n* = 62) sequences in the reference panel was taken as input; an *n* × *n* matrix of all pairwise identities was then produced. The matrix was plotted as a heatmap and scaled such that the maximum observed pairwise identity (100%) was represented by blue and the minimum (97.6%) was represented by red (Fig. 2).

### SNP distribution amongst HPV16 viral lineages

Using Mathematica 7.0 (Wolfram Research, Inc.; see Supplemental Data S2 for code), SNPs were identified by comparing all aligned complete-genome sequences then filtered and grouped according to the lineages [8] for which they were diagnostic, e.g., all SNPs that appeared only in sequences of the E lineage were considered diagnostic for that lineage (Fig. 3, row 2). Similarly, sequences of the E(p) lineage contained all E-specific SNPs, in addition to the E(p)-specific SNPs (Fig. 3, row 1). Clades with HPV16 nucleotide sequences differing by a pairwise value of 1.0% –0.5% were designated as distinct sublineages (e.g., AA1 and AA2); differences of ≥1% were considered variant lineages (e.g., E, Af1, Af2, AA). Lineage-specific SNPs were displayed against position in the genome and color-coded as A (green), C (blue), G (black), T (red) to indicate the nucleotide polymorphism. The plot was positioned adjacent to the Bayesian phylogenetic tree. Location and value of the diagnostic SNPs are listed in Supplemental Table S2.

### Genotype imputation

An imputation algorithm was developed and implemented using a combination of the stand-alone BLAST+ software tools [19] to perform sequence matching (see Supplemental Data S2), and Mathematica 7.0 (Wolfram Research, Inc.) to generate the imputed sequences from the BLAST output (see Supplemental Data S2).

The imputation algorithm consisted of a set of the following actions:

- A BLAST search was performed using 396 partial genome sequences as queries, against a custom BLAST formatted database of the 62 complete-genome reference panel. Hits were constrained to the plus strand only.
- For each partial query sequence, the complete-genome sequence(s) with the highest BLAST scores were selected. In cases where the query sequence was the concatenated sequence of more than one region (i.e., URR and E6), returning separate hits for each region, the complete-genome sequence(s) with the highest combined BLAST score(s) were selected.
- The missing nucleotides of the partial query sequences were completed using the selected best complete-genome sequence.
- In the case of multiple, equally high scoring BLAST hits for a single query sequence, random sampling of the best hits was used by generating a 100 sets of imputed nucleotide sequences. This was done to allow calculation of unbiased confidence intervals for the subsequent odds ratio analysis. Random sampling was performed using the built-in random choice function in Mathematica 7.0 under default options (without replacement sampling based on the ExtendedCA pseudo-random number generator, described in the Mathematica 7.0 accompanying documentation).

### Error characterization

To estimate the precision of imputation with the set of 62 HPV16 reference genomes and the algorithm used herewith, the following analyses were performed. Dummy imputations were conducted using the isolated URR region from each complete-genome sequence in the HPV16 reference panel as a query sequence. The reference panel from which the URR sequences were drawn was used as the BLAST database, under two conditions: with and without removal of the complete HPV16 sequence from which the query sequence was taken. Imputed sequences were then compared to the known complete-genome information at all SNP positions for both methods. For each query sequence, the mean and standard deviation of the percentage of correct SNPs across the best matches for each test sequence was calculated. Averaging the individual means and taking their standard deviation produced overall estimates for each method.

An empirical estimate of imputation error was performed by randomly selecting 8 of the 396 samples (≈2%) that were subject to genotype imputation followed by direct sequencing of their complete genomes. Random selection was performed using the built-in random sample function in Mathematica 7.0 as described above. For each of the 8 samples, the true sequence was compared to 100 imputed sequences. The mean and standard deviation of % error were calculated for each sequenced sample and the overall estimate was calculated by taking the average and standard deviation of the individual means.

### Epidemiological analysis

Of the 35 complete-genome sequences determined from the Guanacaste study and included in the reference database, 16 were also either cases or controls and were combined with the 396 imputed sequences in the epidemiological analyses. Logistic regression was performed on the SNP positions using all imputed sequences and complete genomes in order to compare subjects with CIN3+ to the HPV16-positive “control” population. Only SNP positions with adequate sample size (i.e., ≥10 sequences possessing a SNP; i.e., variant frequency >2.5%) were analyzed. In the logistic regression calculations, the reference group at each position was determined by setting the most prevalent nucleotide as the referent. Logistic regression was run for each of the 100 imputed datasets, parameter estimates and estimated covariances were written to separate datasets. The MIANALYZE procedure was used to combine output data from all analyses. The procedure reads the parameter estimate and covariance computed in each individual logistic regression, and creates a summary estimate and a p-value for each nucleotide position. After Bonferroni correction for 7916 repeated comparisons of SNPs, p-values were considered significant if they were ≤6.32×10^-6^. Odds ratios and 95% confidence intervals were calculated by taking exponentials of the summary estimates. Results were plotted as odds ratios around 1.0, to inspect qualitative trends. All analyses were run using SAS version 9.1.3 (SAS Institute, Cary, NC).

## Supporting Information

Table S1 **Designated names, NCBI accession numbers and lineage assignments for HPV16 isolates.**
(PDF)

Table S2 **Diagnostic single nucleotide polymorphisms (SNPs) for HPV16 lineages.**
(PDF)

Data S1 **Mathematica 7 code for performing imputation based on BLAST results.** A partial sequence of the HPV16 genome was used to search a database of complete genomes using BLAST. The results of the BLAST search were then used to identify the full length genomes that had the highest score and one complete genome sequence was added to a new file that was used for further analyses. Code annotations are enclosed by parentheses and stars, i.e., (*…*). Commands can be pasted directly into a Mathematica notebook and executed. Test data is available from the authors by request.
(PDF)

Data S2 **Mathematica 7 HPV sequence analysis code.** Mathematica 7 notebook containing code used for performing sequence analysis on the nucleotide sequences of a reference HPV complete genome sequence library. Analyses include calculating pairwise differences and plotting a heatmap of values, as well as identifying lineage-specific (diagnostic) single nucleotide polymorphisms. Code annotations are enclosed by parentheses and stars i.e., (*…*). Commands can be pasted directly into a Mathematica notebook and executed. Test data is available from the authors by request.
(PDF)
