# Supplementary material for: Sequence Imputation of HPV16 Genomes for Genetic Association Studies
Source: PLoS One. 2011 Jun 23;6(6):e21375. doi: 10.1371/journal.pone.0021375 (PMC3121793; doi:10.1371/journal.pone.0021375)
Supplement: Table S1 — Designated names, NCBI accession numbers and lineage assignments for HPV16 isolates. (PDF) [file pone.0021375.s001.pdf]

Table S1: Designated names, NCBI accession numbers and lineage assignments for HPV16 isolates.

Isolate[a]	NCBI #	Lineage	GC (%)	Length (nt)
HPV16Ref [b]	NC_001526	E (Prototype 1)	0.365	7906
AS411[b]	HQ644236	E (Prototype 1)	0.366	7905
Qv02234[b]	HQ644259	E (Prototype 1)	0.366	7907
Qv11074[b]	HQ644267	E (Prototype 1)	0.366	7906
Qv11687[b]	HQ644268	E (Prototype 1)	0.366	7904
Qv13956[b]	HQ644271	E (Prototype 1)	0.365	7906
Qv15521[b]	AY686581	E (Prototype 1)	0.366	7906
Qv16936[b]	AY686580	E (Prototype 1)	0.366	7906
Qv17286[b]	HQ644272	E (Prototype 1)	0.365	7902
Qv17722[b]	AY686584	E (Prototype 1)	0.366	7906
Qv18158[b]	AY686583	E (Prototype 1)	0.365	7907
Qv19110[b]	HQ644274	E (Prototype 1)	0.366	7905
Qv24723[b]	HQ644280	E (Prototype 1)	0.366	7905
Qv25054[b]	HQ644282	E (Prototype 1)	0.365	7903
Qv25086[b]	HQ644283	E (Prototype 1)	0.365	7903
Qv28131[b]	HQ644284	E (Prototype 1)	0.367	7905
Qv33501[b]	HQ644286	E (Prototype 1)	0.365	7903
Qv35943[b]	HQ644287	E (Prototype 1)	0.366	7901
Z032[b]	HQ644297	E (Prototype 1)	0.366	7905
W0122[b]	AF536179	E (Prototype 2)	0.368	7904
AS097[b]	HQ644234	E (Asian)	0.366	7905
AS310[b]	HQ644235	E (Asian)	0.366	7905
IN151168[b]	HQ644248	E (Asian)	0.366	7904
INJP0168[b]	HQ644251	E (Asian)	0.366	7904
Qv02706[b]	HQ644261	E (Asian)	0.366	7905
W0724[b]	AF534061	E (Asian)	0.367	7905
BF215[b]	HQ644238	Af-1	0.363	7908
BF325[b]	HQ644240	Af-1	0.363	7908
R872[b]	AF472508	Af-1	0.363	7908
Rw768[b]	HQ644290	Af-1	0.363	7908
Rw918[b]	HQ644293	Af-1	0.363	7908
W0236[b]	AF536180	Af-1	0.364	7906
Z016[b]	HQ644296	Af-1	0.363	7905
Z109[b]	HQ644298	Af-1	0.363	7909
Z122[b]	HQ644299	Af-1	0.363	7905
BF039[b]	HQ644237	Af-2	0.364	7905
BF236[b]	HQ644239	Af-2	0.364	7905

continues on next page

continued from previous page

IN221688[b]	HQ644249	Af-2	0.365	7905
IN272098[b]	HQ644250	Af-2	0.364	7904
R460[b]	AF472509	Af-2	0.365	7904
Rw851[b]	HQ644291	Af-2	0.364	7904
Rw862[b]	HQ644292	Af-2	0.364	7904
Qv00512[b]	HQ644257	Asian-American (NA1)	0.365	7885
IN000078[b]	HQ644247	Asian-American (AA1)	0.365	7906
Qv00079[b]	HQ644253	Asian-American (AA1)	0.365	7910
Qv00346[b]	HQ644255	Asian-American (AA1)	0.365	7908
Qv00995[b]	AF402678	Asian-American (AA1)	0.366	7906
Qv04917[b]	HQ644265	Asian-American (AA1)	0.365	7903
Qv13040[b]	HQ644269	Asian-American (AA1)	0.365	7908
Qv21730[b]	HQ644276	Asian-American (AA1)	0.365	7906
Qv23856[b]	HQ644278	Asian-American (AA1)	0.365	7907
Qv33364[b]	HQ644285	Asian-American (AA1)	0.365	7906
Rw649[b]	HQ644288	Asian-American (AA1)	0.365	7906
Rw677[b]	HQ644289	Asian-American (AA1)	0.365	7906
Qv00124[b]	HQ644254	Asian-American (AA2)	0.366	7907
Qv13791[b]	HQ644270	Asian-American (AA2)	0.366	7909
Qv15321[b]	AY686579	Asian-American (AA2)	0.366	7907
Qv15351[b]	AY686582	Asian-American (AA2)	0.366	7907
Qv19067[b]	HQ644273	Asian-American (AA2)	0.366	7906
Qv22478[b]	HQ644277	Asian-American (AA2)	0.366	7908
Qv23890[b]	HQ644279	Asian-American (AA2)	0.365	7906
Qv24898[b]	HQ644281	Asian-American (AA2)	0.366	7905
Qv00370[c]	HQ644256	E (Prototype 1)	0.366	7906
Qv00930[c]	HQ644258	E (Prototype 1)	0.365	7906
Qv02376[c]	HQ644260	E (Prototype 1)	0.365	7906
Qv03470[c]	HQ644262	E (Prototype 1)	0.365	7906
Qv03874[c]	HQ644264	E (Prototype 1)	0.365	7906
Qv05460[c]	HQ644266	E (Prototype 1)	0.366	7906
Qv20322[c]	HQ644275	E (Prototype 1)	0.365	7905
Qv03545[c]	HQ644263	Asian-American (AA2)	0.366	7906

[a]Isolate number prefix denotes location from which sample was obtained, as follows. AS - Taiwan [1], BF - Burkina Faso [2], IN/INJP - Thailand [3], Qv - Costa Rica [4], R - USA (HAPI study) [5], Rw - Rwanda [6], W - USA (WIHS study) [7], Z - Zambia [8]. [b] These isolates were used as a reference library for sequence imputation. [c] These isolates were randomly selected from amongst 396 partially sequenced HPV16 samples whose sequences were imputed. Missing sequence information for these isolates was also determined through complete HPV16 genome sequencing as described in Materials and Methods. The 8 completely sequenced genomes were used to test the accuracy of the imputation procedure.

References

[1] Liaw KL, Hsing AW, Chen CJ, Schiffman MH, Zhang TY, et al. (1995) Human papillomavirus and cervical neoplasia: a case-control study in taiwan. Int J Cancer 62: 565-71.

[2] Didelot-Rousseau MN, Nagot N, Costes-Martineau V, Vallès X, Ouedraogo A, et al. (2006) Human papillomavirus genotype distribution and cervical squamous intraepithelial lesions among high-risk women with and without hiv-1 infection in burkina faso. Br J Cancer 95: 355-62.

[3] Marks M, Gupta SB, Liaw KL, Kim E, Tadesse A, et al. (2009) Confirmation and quantitation of human papillomavirus type 52 by roche linear array using hpv52-specific taqman e6/e7 quantitative real-time pcr. J Virol Methods 156: 152-6.

[4] Schiffman M, Herrero R, Desalle R, Hildesheim A, Wacholder S, et al. (2005) The carcinogenicity of human papillomavirus types reflects viral evolution. Virology 337: 76-84.

[5] Ho GY, Bierman R, Beardsley L, Chang CJ, Burk RD (1998) Natural history of cervicovaginal papillomavirus infection in young women. New England Journal of Medicine 338: 423-428.

[6] Singh DK, Anastos K, Hoover DR, Burk RD, Shi Q, et al. (2009) Human papillomavirus infection and cervical cytology in hiv-infected and hiv-uninfected rwandan women. J Infect Dis 199: 1851-61.

[7] Strickler HD, Palefsky JM, Shah KV, Anastos K, Klein RS, et al. (2003) Human papillomavirus type 16 and immune status in human immunodeficiency virus-seropositive women. J Natl Cancer Inst 95: 1062-71.

[8] Sahasrabuddhe VV, Mwanahamuntu MH, Vermund SH, Huh WK, Lyon MD, et al. (2007) Prevalence and distribution of hpv genotypes among hiv-infected women in zambia. Br J Cancer 96: 1480-3.
