# Supplementary material for: Sequence Imputation of HPV16 Genomes for Genetic Association Studies
Source: PLoS One. 2011 Jun 23;6(6):e21375. doi: 10.1371/journal.pone.0021375 (PMC3121793; doi:10.1371/journal.pone.0021375)
Supplement: Table S2 — Diagnostic single nucleotide polymorphisms (SNPs) for HPV16 lineages. (PDF) [file pone.0021375.s002.pdf]

Table S2: Diagnostic single nucleotide polymorphisms (SNPs) for HPV16 lineages.

Lineage	Diagnostic SNPs[a]
**E(p)**	1842A, 3159C, 3249G, 3787C
**E**	145G, 286T, 289A, 335C, 789T, 1096C, 1366T, 1377C, 1486T, 2041C, 2220G, 2344C, 2586T, 2631T, 2860C, 3182G, 3362A, 3377C, 3516C, 3566T, 3694T, 3778G, 3858T, 4089T, 4157A, 4290T, 4437G, 4609T, 4653T, 5151G, 5573C, 5707G, 5873C, 5920T, 6174C, 6256T, 6568C, 6730G, 6863C, 6979C, 7498G, 7773C, 7795C
**E(As)**	24T, 178G, 846C, 3524C, 4077T, 7184C, 7186C, 7210C, 7279T, 7296C, 7851A
**Af-1**	3868A, 4042T, 4145T, 4317A, 4437A, 4920T, 5668C, 6577A, 7241G, 7885A
**Non-E**	145T, 286A, 289G, 335T, 789C, 795G, 1096G, 1366A, 1377T, 1486C, 2041T, 2220C, 2344T, 2586C, 2631A, 2860A, 3182A, 3362G, 3377G, 3516A, 3566G, 3694A, 3778T, 3858C, 4089C, 4157G, 4158A, 4176T, 4290C, 4609C, 4653A, 5151A, 5388A, 5398A, 5412C, 5504C, 5515A, 5573G, 5707A, 5873T, 5920C, 6174A, 6256C, 6568T, 6730A, 6863T, 6979T, 7069T, 7498A, 7773T, 7795T
**Af-2**	109C, 132T, 403G, 1416T, 3043T, 3431A, 4149C, 4169G, 4202C, 4527G, 4554G, 4863T, 5268G, 5299C, 6491C, 7444A, 7835A, 7846C, 7848G
**NA1/AA/Af-2**	1163A, 1200C, 1744A, 2237G, 2249A, 2262T, 2287T, 3517C, 3538C, 3706C, 3805G, 4437T, 4896G, 5496T, 6704C, 6874T, 7242C, 7494C, 7678T
**NA1**	1221C, 2253C, 3371C, 3413A, 3838C, 4585G, 5082T, 5161C, 5541G, 6182A, 7030T, 7236C
**NA1/AA**	1668G, 3224A, 4017A, 4151A, 4177T, 4461C, 4959G, 4978G, 5043T, 5295A, 5377G, 5395G, 7738C
**AA2**	3387C, 4608C, 5304C, 5319A, 5484A, 6812T, 7752G
**AA**	732C, 1041G, 2343C, 2650A, 4180A, 4201C, 4215G, 4953G, 7348T, 7404T
**AA1**	1006C, 1862G, 3226G, 3244C, 3416A, 3967G, 4213C, 4419G, 5296C, 6034C, 7516G

[a]Diagnostic nucleotides were determined by finding the sets of nucleotides that were unique to each HPV16 lineage. The code for this process can be found in Supplementary Data S2. The results are displayed graphically in Figure 3 of the manuscript. Nucleotide positions are given relative to the HPV16 reference sequence (NCBI# NC_001526).
