# Supplementary material for: Sequence Imputation of HPV16 Genomes for Genetic Association Studies
Source: PLoS One. 2011 Jun 23;6(6):e21375. doi: 10.1371/journal.pone.0021375 (PMC3121793; doi:10.1371/journal.pone.0021375)
Supplement: Data S1 — Mathematica 7 code for performing imputation based on BLAST results. A partial sequence of the HPV16 genome was used to search a database of complete genomes using BLAST. The results of the BLAST search were then used to identify the full length genomes that had the highest score and one complete genome sequence was added to a new file that was used for further analyses. Code annotations are enclosed by parentheses and stars, i.e., (*…*). Commands can be pasted directly into a Mathematica notebook and executed. Test data is available from the authors by request. (PDF) [file pone.0021375.s003.pdf]

Importing aligned sequence data

```
infile = SystemDialogInput["FileOpen",
   "/Users/bensmith/Documents/Ben/HPV16 Project 7.09/",
   WindowTitle → "Select .fasta alignemnet file to open..."];
 (*Stores the alignment file filepath as a variable*)
seqdata = Import[infile];
(*Imports the sequences as a list of strings from the alignment file*)
namedata = Import[infile, "Header"];
(*Imports the sequence names (Headers) as a list of strings from
 the alignment file*)
```

Ordering sequences for publication

```
order = {18, 20, 21, 19, 17, 22, 23, 24, 25, 13, 14, 15, 16, 11, 12, 10,
   9, 8, 26, 7, 1, 6, 5, 4, 2, 3, 57, 60, 58, 59, 61, 56, 55, 54, 62, 29,
   28, 27, 32, 33, 31, 30, 34, 37, 40, 43, 42, 35, 38, 41, 39, 44, 36, 45,
   46, 47, 48, 52, 50, 49, 51, 53};
```

Formatting sequence data

```
cgseqsuo = Characters[#] & /@
   seqdata[[
    Flatten[Position[namedata, #] & /@
      Flatten[StringCases[namedata, StartOfString ~~ "HPV16|" ~~ __]]]]];
 (*Selects the complete genome sequences by looking in the names
 list and finding the positions corresponding to any starting with "HPV16|",
and stores them in a seperate list*)
cgseqs = cgseqsuo[[order]];(*orders sequences to match phylogenetic tree*)
cgsamplenamesuo =
  Flatten[StringCases[namedata, StartOfString ~~ "HPV16|" ~~ __]];
 (*Stores the full names in a seperate list*)
cgsamplenames = cgsamplenamesuo[[order]];
(*orders the list of names to match the order in cgseqs*)
cglabIDs = Part[StringSplit[#1, "|"], 2] & /@ cgsamplenames
(*Spilts the full name up and stores the seperate parts*)
cglineages = Part[StringSplit[#1, "|"], 3] & /@ cgsamplenames
cgstatusfromname =
  DeleteCases[
   If[Length[ StringSplit[#1, "|"]] == 4,
      {Part[StringSplit[#1, "|"], 2], Part[StringSplit[#1, "|"], 4]}] & /@
    cgsamplenames, Null];
cgIDlin =
 StringJoin[Part[StringSplit[#1, "|"], 2], "|",
    Part[StringSplit[#1, "|"], 3]] & /@ cgsamplenames
(*Joins the lab ID and the lineage for each sequence*)
posbase = Table[{i, {j, cgseqs[[i, j]]}}, {i, Length[cgseqs]},
   {j, Length[cgseqs[[1]]]}];
 (*Takes each nucleotide in each sequence and associates it with
 a numerical sequence i.d. and a position*)
cgposbase = posbase[[All, All, 2]];
(*Stris the sequence index from the "posbase" list"*)
cglength = Length[cgseqs[[1]]];
(*Gets the length of the alignment*)

TableForm[Transpose[{cglabIDs, cglineages}], TableHeadings → Automatic]
```

Reference panel analysis

```
pwcomp =
  Table[100 N[Length[Complement[cgposbase[[i]], cgposbase[[j]]]] / cglength],
   {i, 1, Length[cgposbase]}, {j, 1, Length[cgposbase]}];
 (*Counts the number of different nucleotides between every pair
 of sequences*)
pwdiff = Table[ N[Length[Complement[cgposbase[[i]], cgposbase[[j]]]]],
   {i, 1, Length[cgposbase]}, {j, 1, Length[cgposbase]}];
```

```
pwcompplot = ArrayPlot[(100 - pwcomp), Mesh → All,
  ColorFunction →
   (If[# < 97.6, GrayLevel[.5],
      Hue[(5 / 8) * ((# - Min[100 - Flatten[pwcomp]]) /
           (Max[100 - Flatten[pwcomp]] - Min[100 - Flatten[pwcomp]]))]] &),
  ColorFunctionScaling → False, ImageSize → 1000, Frame → True,
  FrameTicks → None](*Plots the SNP comparison table,
 colouring the surface according to identity*)

pwcomptabplot =
 TableForm[
  Table[
   Tooltip[
    Graphics[{EdgeForm[{Thin, Black}],
      Apply[Hue,
       {((5 / 8) *
          (1 - ((pwcomp[[i, j]] - Min[Flatten[pwcomp]]) /
              (Max[Flatten[pwcomp]] - Min[Flatten[pwcomp]]))))}],
      Rectangle[]}, ImageSize → 15],
    Flatten[{cgIDlin[[i]], "vs", cgIDlin[[j]], "=",
      NumberForm[(100 - pwcomp[[i, j]]), {4, 2}], "%"}]],
   {i, 1, Length[cgIDlin]}, {j, 1, Length[cgIDlin]}], TableSpacing → {0, 0},
  TableHeadings →
   {Flatten[cgIDlin], Rotate[#1, 90 Degree] & /@ Flatten[cgIDlin]}]
```

Grouping nucleotides

```
groups =
   Sort[Gather[Gather[Select[Flatten[posbase, 1], # ≠ {_, {_, "-"}} &],
      #1[[2]] == #2[[2]] &], #1[[All, 1]] == #2[[All, 1]] &],
    Length[#1] > Length[#2] &];;
(*Grouping and selection function. First groups all identical
 (position, nucleotide) pairs. Then groups those nucleotides that
 are associated with the same sets of sequences. Finally,
sorts these groupings so that those with the largest number of
 group defining nucleotides are earlier.*)
(*potentialerrors=Select[groups,Part[Dimensions[#],1;;2]=={1,1}&]
novelseqs=Select[groups,Part[Dimensions[#],2]<2&]*)
```

```
lineageindexes = {{1, 20}, {1, 26}, {21, 26}, {27, 35}, {36, 42}, {27, 62},
   {36, 62}, {43, 43}, {43, 62}, {44, 54}, {44, 62}, {55, 62}};
liorder = {1, 2, 3, 4, 6, 5, 7, 8, 9, 10, 11, 12};
lineageindexes = lineageindexes[[liorder]];
names = {{"Ep"}, {"E"}, {"E.As"}, {"Af1"}, {"Af2"}, {"Non-E"},
   {"NA1/AA/Af2"}, {"NA1"}, {"NA1/AA"}, {"AA2"}, {"AA"}, {"AA1"}};
names = names[[liorder]];
lsSNPs =
  Table[
   Flatten[Select[Rest[groups],
      #[[1, All, 1]] == Range[lineageindexes[[i, 1]],
         lineageindexes[[i, 2]]] &], 1][[All, 1, 2]],
   {i, Length[lineageindexes]}];
nlsSNPs = {"N", {Length[lsSNPs] + 1, #[[1]]}} & /@
   DeleteDuplicates[Complement[Flatten[Rest[groups], 1][[All, 1, 2]],
     Flatten[lsSNPs, 1]], #1[[1]] == #2[[1]] &];
```

Visualising SNP distribution

```
prearray = Table[{#[[2]], {i, #[[1]]}} & /@ lsSNPs[[i]], {i, Length[lsSNPs]}];
(*creates a table where the lineage of the lineage specific SNP
 positions and the lineage index are in a list. These lists are
 nested within another list containing the nucleotide value of
 the SNP. This is done to facilitate later sorting*)
groupedprearray = Gather[Flatten[prearray, 1], #1[[1]] == #2[[1]] &];
(*This places lineage specific SNPs into 4 groups,
each group corresponding to a nucleotide value. The order is A,
C, G, T, N.*)
array = #[[All, 2]] & /@ groupedprearray;
(*Selects just the (index, position) pais from "groupedprearray".*)

linfixplot =
 Rotate[ListPlot[Tooltip[array], PlotStyle → Black,
   FillingStyle → Directive[Opacity[0.8], Gray], Axes → {False, True},
   AxesLabel → {"Sequence Group", "Position"},
   FrameTicks →
    {Table[{i, Rotate[names[[i]], 90 Degree]}, {i, Length[lsSNPs]}],
     Table[{i, Rotate[Text[Style[i, Black, 24]], 180 Degree]},
      {i, 0, 8000, 1000}]}, PlotRange → {{0, Length[lsSNPs] + 1}, {0, 8000}},
   AxesOrigin → {0, 0},
   PlotMarkers →
    {Text[Style["—", Darker[Green], Bold, 16]],
     Text[Style["—", Blue, Bold, 16]], Text[Style["—", Black, Bold, 16]],
     Text[Style["—", Red, Bold, 16]], Text[Style["—", Gray, Bold, 16]]},
   GridLines → {Table[i, {i, Length[lsSNPs]}], None},
   Frame → {{False, True}, {False, False}}, ImageSize → 500, AspectRatio → 2],
  270 Degree]
```
