# Supplementary material for: Sequence Imputation of HPV16 Genomes for Genetic Association Studies
Source: PLoS One. 2011 Jun 23;6(6):e21375. doi: 10.1371/journal.pone.0021375 (PMC3121793; doi:10.1371/journal.pone.0021375)
Supplement: Data S2 — Mathematica 7 HPV sequence analysis code. Mathematica 7 notebook containing code used for performing sequence analysis on the nucleotide sequences of a reference HPV complete genome sequence library. Analyses include calculating pairwise differences and plotting a heatmap of values, as well as identifying lineage-specific (diagnostic) single nucleotide polymorphisms. Code annotations are enclosed by parentheses and stars i.e., (*…*). Commands can be pasted directly into a Mathematica notebook and executed. Test data is available from the authors by request. (PDF) [file pone.0021375.s004.pdf]

Import and parse reference database sequences, query sequences and BLAST results

In[108]:=
```
myDBfile = SystemDialogInput["FileOpen",
  WindowTitle →
   "Select FASTA format file from which custom BLAST database was
     created..."](*Set FASTA format file containing BLAST
  database sequences*)
```

Out[108]=
```
/Users/bensmith/Documents/Ben/2009.07
  Imputation Project/Guanacaste prediction/62CG.fasta
```

In[109]:=
```
myDBseqs = Import[myDBfile, "Sequence"];
myDBheads = Import[myDBfile, "Header"];
(*Import database sequences and headers*)
```

In[111]:=
```
myQueryfile = SystemDialogInput["FileOpen",
  WindowTitle → "Select FASTA format query file..."]
  (*Set FASTA format file containing BLAST query sequences*)
```

Out[111]=
```
/Users/bensmith/Documents/Ben/2009.07
  Imputation Project/Guanacaste prediction/QvQueries.fasta
```

In[112]:=
```
myQueryseqs = Import[myQueryfile, "Sequence"];
myQueryheads = Import[myQueryfile, "Header"];
(*Import query sequences and headers*)
```

In[114]:=
```
myInfile = SystemDialogInput["FileOpen",
  WindowTitle → "Select BLAST results file for analysis..."]
(*Set BLAST result filename for import*)
```

Out[114]=
```
/Users/bensmith/Documents/Ben/2009.07
  Imputation Project/Guanacaste prediction/out.Qv.txt
```

In[115]:=
```
myHitlines = StringSplit[#] & /@
   Flatten[StringCases[StringSplit[Import[myInfile], "\n"],
     StartOfString ~~ "Qv" ~~ __]];
 (*Import raw BLAST output file and select lines containing hit
 data and store as a list of lists*)
```

In[116]:=
```
myBLASThits = Gather[myHitlines, #1[[1]] == #2[[1]] &];
(*Separate "myHitlines" so that each query result is contained
 in it's own list*)
```

In[117]:=
```
myNohitqueries = Complement[myQueryheads, myBLASThits[[All, 1, 1]]]
(*Determines which, if any, query sequences produced no BLAST hits*)
myNohitpos = Flatten[Position[myQueryheads, #] & /@ myNohitqueries, 1];
(*Finds the positions in the query list of those queries that
 produced no BLAST results*)
```

Out[117]=
```
{Qv03333, Qv17829, Qv17973}
```

In[119]:=
```
(*The following excludes a list of query sequences that are in
 the myQueryfile sequence file. Excluded sequences are those that
 returned no BLAST hits and those from an imported CSV format file
 specifying additional names of queries to be excluded. If no
 exclude file is chosen it will only exclude queries with no BLAST
 hits. If all queries produced hits and no exclude file is chosen,
nothing is excluded*)
myQueryseqs = Delete[myQueryseqs, myNohitpos];
myQueryheads = Delete[myQueryheads, myNohitpos];
(*Excludes query sequences that returned no BLAST hits from further
 analysis. These lists now match the "myBLASThits" list in terms
 of length and positions of query sequences*)
myExcludesfile =
 Quiet[SystemDialogInput["FileOpen",
   WindowTitle → "Select CSV format exclude list..."]];
(*Set CSV format file containing additional Query sequences to exclude*)
myQueryexcludes = Quiet[Flatten[Import[myExcludesfile, "CSV"]]];
(*Import list of query sequence names to exclude*)
myExcludepositions =
  Quiet[Flatten[Position[myQueryheads, #] & /@ myQueryexcludes, 1]];
 (*Returns a position list of query sequences to exclude from
 further analysis*)
myBLASThits = Delete[myBLASThits, myExcludepositions];
myQueryseqs = Delete[myQueryseqs, myExcludepositions];
myQueryheads = Delete[myQueryheads, myExcludepositions];
(*Excludes the specified additional query sequences from the
 neccessary lists of data*)
```

Create functions that return information about a sepcified query sequence

```
gatheredHits[i_] := Gather[myBLASThits[[i, All]], #1[[2]] == #2[[2]] &];
(*Function to group hits corresponding to fragments of the query
 sequence that recognize different parts of the same sequence,
for a specified query sequence given as an integer argument*)
listCombinedScores[i_] :=
  Table[Total[ToExpression[gatheredHits[i][[j, All, 4]]]],
   {j, Length[gatheredHits[i]]}];
(*Function to produce a list of the summed scores from multiple
 hits of different parts of the query sequence,
for a specified query sequence given as an integer argument*)

bestHits[i_] :=
 gatheredHits[
   i][[Flatten[Position[listCombinedScores[i], Max[listCombinedScores[i]]]],
   All]](*Selects the best hits for a specified query sequence
 based on the combined scores,
for a specified query sequence given as an integer argument*)
bestHitPos[i_] :=
 Flatten[ToExpression[StringCases[bestHits[i][[All, 1, 2]],
    StartOfString ~~ DigitCharacter ..]]]
(*Returns position in "myDBseqs" and "myDBheads" of best hits,
for a specified query sequence given as an integer argument*)
bestHitNames[i_] := myDBheads[[bestHitPos[i]]]
(*Returns the sequence header information of the best hits,
for a specified query sequence given as an integer argument*)
bestHitSeqs[i_] :=
 myDBseqs[[Flatten[
    ToExpression[StringCases[bestHits[i][[All, 1, 2]],
      StartOfString ~~ DigitCharacter ..]]]]]
  (*Returns the best hit sequences from "myDBseqs",
for a specified query sequence given as an integer argument*)
bestHitQpos[i_] := ToExpression[#] & /@ bestHits[i][[All, All, 5 ;; 6]]
(*Returns the start and end positions of the query sequence in
 the best hits, for a specified query sequence given as an integer
 argument *)
bestHitSpos[i_] := ToExpression[#] & /@ bestHits[i][[All, All, 7 ;; 8]]
(*Returns the start and end positions of the best hit sequences,
```

```
for a specified query sequence given as an integer argument*)
bestHitQseqs[i_] := StringTake[myQueryseqs[[i]], #] & /@
  bestHitQpos[i][[All, All]]
  (*Returns the parts of the query sequence used in the best hits,
for a specified query sequence given as an integer argument*)
imputedSeqs[i_] :=
 Apply[StringReplacePart,
  Transpose[{bestHitSeqs[i], bestHitQseqs[i], bestHitSpos[i]}], {1}]
  (*Replaces the sections in the best hit sequences with the
 corresponding BLAST aligned segments of the query sequence,
returning a list of possible imputed complete genome sequences
 for the query sequence specified as an integer argument*)
randomImputedSeqs[i_, n_] := RandomChoice[imputedSeqs[i], n]
(*Returns n imputed complete genome sequences,
pseudo-randomly chosen from the possibilities according to best BLAST hits,
for the ith query sequence. i is specified by the first argument,
n is specified by the second.*)
allImputedSeqs[n_] :=
 Transpose[randomImputedSeqs[#, n] & /@ Range[Length[myQueryseqs]]]
 (*Returns n sets of imputed sequences,
 with each set containing an imputed sequence for every query
  sequence. Each of the imputed sequences are chosen at random
  from amongst the best matches for each query sequence*)
```

Export imputed complete genome sequences

```
Do[Export[StringJoin["/Users/home/Desktop/ImputedSeqs/",
   myQueryheads[[j]], ".fasta"],
  {Table[StringJoin[" Match ", ToString[i], ": ", bestHitNames[j][[i]]],
    {i, Length[imputedSeqs[j]]}], imputedSeqs[j]}, "FASTA"],
 {j, Length[myQueryheads]}]
 (*Exports seperate FASTA format files for each query sequence
 containing its best BLAST hits*)

n = Input["Enter number of imputation samplings to perform"];
Do[Export[StringJoin["/Users/home/Desktop/ImputedSeqs/Imputation",
   ToString[j], ".fasta"],
  {Table[myQueryheads[[i]], {i, Length[myQueryheads]}],
   allImputedSeqs[n][[j]]}, "FASTA"], {j, n}]
n =. (*Exports each imputation sample as a seperate file containing
 an imputed sequence for each query sequence*)
```
